# Poor bone mineral density aggravates adjacent segment's motility compensation in patients with oblique lumbar interbody fusion with and without pedicle screw fixation: An *in silico* study

**DOI:** 10.3389/fsurg.2022.967399

**Published:** 2022-08-31

**Authors:** Chen-Yi Huang, Zi-Fan Zhang, Xiao-Yu Zhang, Fei Liu, Zhong-Xin Fang, Zhi-Peng Xi, Jing-Chi Li

**Affiliations:** ^1^ Department of Orthopedics, Hospital (T.C.M) Affiliated to Southwest Medical University, Luzhou, China; ^2^ Department of Spine Surgery, Shanghai Changzheng Hospital, Naval Medical University, Shanghai, China; ^3^ Department of Orthopedics, Affiliated Hospital of Integrated Traditional Chinese and Western Medicine for Nanjing University of Chinese Medicine, Nanjing, China; ^4^ Fluid and Power Machinery Key Laboratory of Ministry of Education, Xihua University, Chengdu, China

**Keywords:** adjacent segment diseases, oblique lumbar interbody fusion, motility compensation, bone mineral density, finite elemant analysis

## Abstract

**Objective:**

Motility compensation increases the risk of adjacent segment diseases (ASDs). Previous studies have demonstrated that patients with ASD have a poor bone mineral density (BMD), and changes in BMD affect the biomechanical environment of bones and tissues, possibly leading to an increase in ASD incidence. However, whether poor BMD increases the risk of ASD by aggravating the motility compensation of the adjacent segment remains unclear. The present study aimed to clarify this relationship in oblique lumbar interbody fusion (OLIF) models with different BMDs and additional fixation methods.

**Methods:**

Stand-alone (S-A) OLIF and OLIF fixed with bilateral pedicle screws (BPS) were simulated in the L4–L5 segment of our well-validated lumbosacral model. Range of motions (ROMs) and stiffness in the surgical segment and at the cranial and caudal sides’ adjacent segments were computed under flexion, extension, and unilateral bending and axial rotation loading conditions.

**Results:**

Under most loading conditions, the motility compensation of both cranial and caudal segments adjacent to the OLIF segment steeply aggravated with BMD reduction in S-A and BPS OLIF models. More severe motility compensation of the adjacent segment was observed in BPS models than in S-A models. Correspondingly, the surgical segment's stiffness of S-A models was apparently lower than that of BPS models (S-A models showed higher ROMs and lower stiffness in the surgical segment).

**Conclusion:**

Poor BMD aggravates the motility compensation of adjacent segments after both S-A OLIF and OLIF with BPS fixation. This variation may cause a higher risk of ASD in OLIF patients with poor BMD. S-A OLIF cannot provide instant postoperative stability; therefore, the daily motions of patients with S-A OLIF should be restricted before ideal interbody fusion to avoid surgical segment complications.

## Introduction

Lumbar interbody fusion (LIF) surgeries are widely used to treat lumbar degenerative diseases (LDDs) (1, 2). Adjacent segment diseases (ASDs) are a common complication of spinal fusion surgery (3, 4). Motility compensation is an essential mechanism of biomechanical deterioration of the adjacent segment (5, 6). The stiffness of the interbody cage and grafted bone (GB) is higher than that of intervertebral disc (IVD) components. During LIF surgeries, the nucleus, cartilage endplates (CEPs), and parts of the annulus are replaced by the cage and GB (1, 7). Thus, the fusion segment shows higher stiffness than the original IVD. Consequently, the stiffness of the fusion segment is increased, and its range of motions (ROMs) is decreased under the same moments. ROMs of adjacent segments must be increased to achieve similar ROMs of the lumbar spine in different body positions (5, 6). This pathological process increases the risk of accelerated disc degeneration (DD) and instability in adjacent segments, leading to a poor prognosis for LIF patients (3, 4).

As mentioned above, biomechanical deterioration leads to an increased risk of developing ASDs (8, 9). According to surgeons, the demographic characteristics of patients with ASDs are closely related to certain types of biomechanical deterioration. Specifically, clinical follow-up studies have shown that patients with high body mass index (BMI) have a higher incidence of ASDs; correspondingly, biomechanical studies have confirmed that overweight patients have higher intradiscal pressure and annulus shear stress, which leads to annulus tear risk (3, 4). Elderly patients are at a greater risk of developing ASD; correspondingly, preexisting DD is confirmed as a risk factor for annulus stress concentration and further acceleration of DD (5, 10). Clinical studies have also shown that patients with osteoporosis have a higher risk of DD and ASD, but the biomechanical significance of poor bone mineral density (BMD) remains unclear (3, 4, 11).

Our previous study showed that poor BMD leads to stress concentration in adjacent segments; however, Zhang et al. reported a contrasting finding by using an approximate research method (8, 12). The indicator selected in both these studies was, however, limited to the stress distribution of IVDs, and there was a lack of explanation of how changes in BMD affect the motility compensation of the adjacent segment. Additional fixation devices (AFDs) are also commonly used to provide instant stability to the LIF segment (13, 14). The bilateral pedicle screw (BPS) is an extensively used AFD. Although BPS removal after interbody bone integration will alleviate biomechanical deterioration of the adjacent segment (5, 6, 15), no study has assessed whether the use of BPS aggravates motility compensation of the adjacent segments in the early postoperative period as compared to the stand-alone (S-A) surgical method (i.e., LIF without any AFD fixation).

On the basis of the abovementioned theoretical and practical knowledge, we hypothesize that poor BMD may cause a high risk of ASD by aggravating pathological motility compensation of the adjacent segment. To confirm this hypothesis, we simulated S-A oblique lumbar interbody fusion (OLIF) and OLIF with BPS fixation in well-validated finite element (FE) models with different BMDs. ROMs and stiffness in both surgical and adjacent segments were computed and recorded to identify surgical segment stability and motility compensation of the adjacent segments.

## Methods

### Model construction and validation

We simulated S-A OLIF and OLIF with BPS fixation in a well-validated FE lumbosacral model. In this process, we performed a multi-indicator model validation to verify the computational credibility of the FE model (16, 17). To construct bony structures, reconstructed bony outlines were inputted into 3D CAD software, and outlines of bony structures were drawn by fitted curves to construct bony structures with fitted surfaces. In this process, bony structures, including cortical, cancellous, and bony endplates, were constructed separately. The cortical thickness was set to 0.5 mm, and the thickness, concave angles, and depth in both coronal and sagittal planes were defined according to the measurements of imaging data and anatomical samples (18–20). Nonbony components were constructed in the same 3D CAD software. IVD components comprise the annulus, nucleus, and CEPs, and the outline of the CEP covers the nucleus and inner parts of the annulus (21, 22). Facet cartilages were defined as contact-to-contact surfaces, and ligament structures were defined as cable elements (23, 24). To validate whether the current model represents actual biomechanical situations, computed intradiscal pressure, facet contact force, disc compression value, and different directional ROMs were calculated and compared with the average values of the indicators recorded in in-vitro tests. Given that the differences between the computed and tested values were less than one standard deviation, we believe that the current model adequately represents actual biomechanical situations and can be used in current surgical simulations.

### Surgical simulations

**Figure 1:**
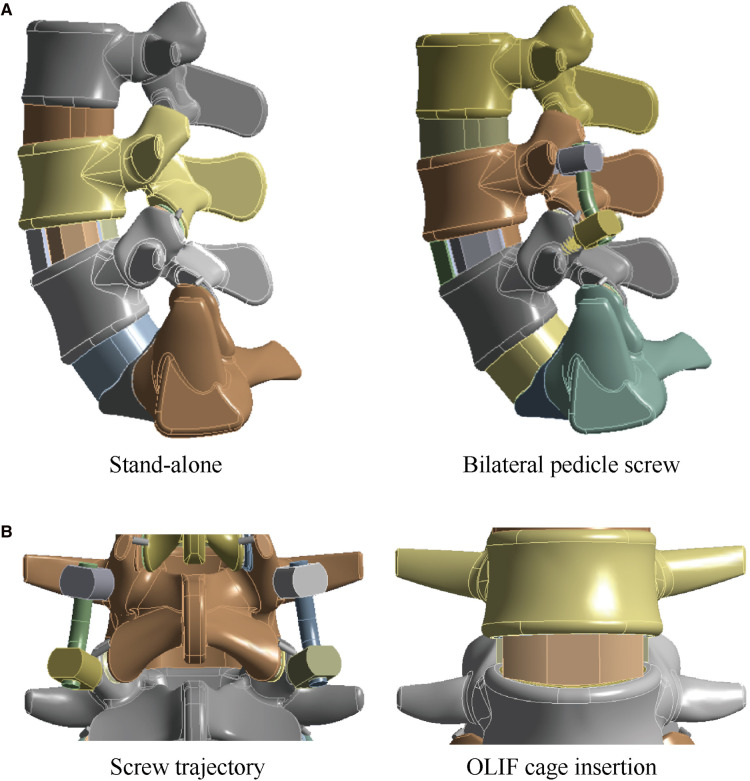
Schematic for surgical simulations in the lumbo-sacral model. (**A**) The intact lumbo-sacral model; (**B**) Simulations for S-A OLIF and OLIF fifixed by BPS.

We performed OLIF simulations in the L4–L5 IVD because of the highest incidence of LDDs in this motion segment. The length of the OLIF cage was defined according to the measurement of vertebral body sizes. An OLIF cage model of 50 mm length was constructed in the same 3D CAD software. The nucleus, CEPs, and two lateral sides of the annulus were removed to simulate the discectomy and endplate preparation, and the OLIF cage fully covered with GB was inserted into the interbody space (Figure 1) (25, 26). The long axis of the OLIF cage was parallel to the coronal plane of the lumbosacral models. The height of the interbody space and lordotic angles of the surgical segment were kept identical to the corresponding postoperative models to eliminate their biomechanical effects (26, 27). S-A OLIF simulations were accomplished by performing these procedures. For simulating percutaneous BPS fixation, cannulated pedicle screw models of 6.5 mm diameter were constructed. Four identical pedicle screws were inserted into the L4 and L5 vertebral bodies (Figure 1) (28, 29).

### Boundary and loading conditions

The boundary and loading conditions of the current models were defined according to *in vitro* biomechanical tests. The inferior surfaces of S1 were completely fixed, while different directional moments were applied to the superior surfaces of L3 (8, 21). Models were computed under identical loading conditions, including 8 Nm flexion, 6 Nm extension and bending, and 4 Nm axial rotation (30, 31). Because current models are symmetrical in the central sagittal plane alone, bending and axial rotation loading conditions can be computed unilaterally. The contact between facet cartilages was set as frictionless. The frictional coefficient between the OLIF cage and BEPs was set as 0.2, and that between the GB and BEPs was set as 0.46 to simulate the instant postoperative biomechanical environment (32, 33).

Mesh generation strategies used in the present study were consistent with those reported in our previous studies, and the mesh convergence test was also performed to eliminate the effect of mesh size on the biomechanical performance of the models (16, 17). The annulus was defined as a hypoelastic material, and the nucleus was set as a semifluid incompressible bag (15, 34). Pedicle screw material was defined as titanium alloy (Ti6Al4 V), and the OLIF cage was defined as polyether ether ketone (PEEK); the elastic modulus of the GB was calculated based on the measurement of Hounsfield unit (HU) values immediately after the CT scan (34, 35). The material properties of cortical and cancellous bones were defined according to anisotropic laws, and BEP was set as an isotropic material (36, 37). For constructing postoperative models with normal BMD, osteopenia, and osteoporosis, the stiffness of cortical, cancellous, and BEPs was adjusted according to the same numerical simulations and tests used for bony material properties (Table 1). The morphological parameters of bony structures remained unchanged (37–39).

**Table 1:** Material properties of FE models’ components.

Components	Elastic modulus (MPa)	Poisson's ratio	Cross-section (mm^2^)
Cortical (normal BMD)	E_xx _= 11,300	V_xy _= 0.484	
	E_yy _= 11,300	V_yz _= 0.203	
	E_zz _= 22,000	V_xz _= 0.203	
	G_xy _= 3,800		
	G_yz _= 5,400		
	G_xz _= 5,400		
Cancellous (normal BMD)	E_xx _= 140	V_xy _= 0.45	
	E_yy _= 140	V_yz _= 0.315	
	E_zz _= 200	V_xz _= 0.315	
	G_xy _= 48.3		
	G_yz _= 48.3		
	G_xz _= 48.3		
Bony endplates (normal BMD)	12,000	0.3	
Cortical (slight reduction of BMD)	Exx = 9,436	Vxy = 0.484	
	Eyy = 9,436	Vyz = 0.203	
	Ezz = 18,370	Vxz = 0.203	
	Gxy = 3,173		
	Gyz = 4,509		
	Gxz = 4,509		
Cancellous (slight reduction of BMD)	Exx = 93.8	Vxy = 0.45	
	Eyy = 93.8	Vyz = 0.315	
	Ezz = 150	Vxz = 0.315	
	Gxy = 32.36		
	Gyz = 36.23		
	Gxz = 36.23		
Bony endplates (slight reduction of BMD)	10,035	0.3	
Cortical (significant reduction of BMD)	Exx = 7,571	Vxy = 0.484	
	Eyy = 7,571	Vyz = 0.203	
	Ezz = 14,740	Vxz = 0.203	
	Gxy = 2,546		
	Gyz = 3,618		
	Gxz = 3,618		
Cancellous (significant reduction of BMD)	Exx = 47.6	Vxy = 0.45	
	Eyy = 47.6	Vyz = 0.315	
	Ezz = 100	Vxz = 0.315	
	Gxy = 16.42		
	Gyz = 24.15		
	Gxz = 24.15		
Bony endplates (significant reduction of BMD)	8,070	0.3	
Annulus	Hypoelastic material		
Nucleus	1	0.49	
Cartilage endplates	10	0.4	
Anterior longitudinal ligaments	Calibrated load-deformation curved under different loading conditions	0.3	60
Posterior longitudinal ligaments	Calibrated load-deformation curved under different loading conditions	0.3	21
Ligamentum flavum	Calibrated load-deformation curved under different loading conditions	0.3	60
Interspinous ligaments	Calibrated load-deformation curved under different loading conditions	0.3	40
Supraspinous ligaments	Calibrated load-deformation curved under different loading conditions	0.3	30
Intertransverse ligaments	Calibrated load-deformation curved under different loading conditions	0.3	10
Capsular	7.5 (25%)	0.3	67.5
	32.9 (25%)		
PEEK OLIF cage	3,500	0.3	
Titanium alloy screw	110,000	0.3	

## Results

### Motility compensation of adjacent segments in models with different BMDs

**Figure 2:**
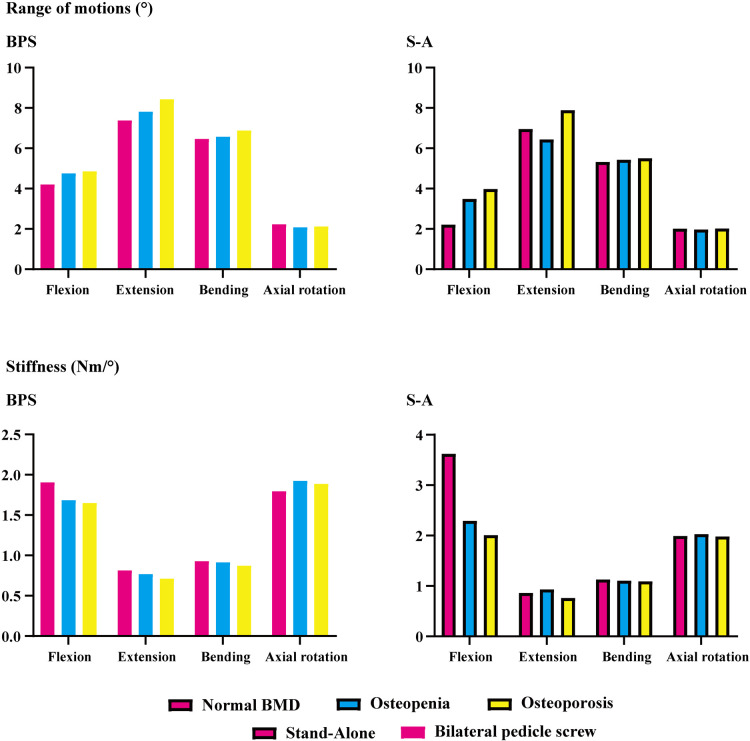
ROMs and stiffness of the motion segment cranial to the surgical segment (L3–L4).

**Figure 3:**
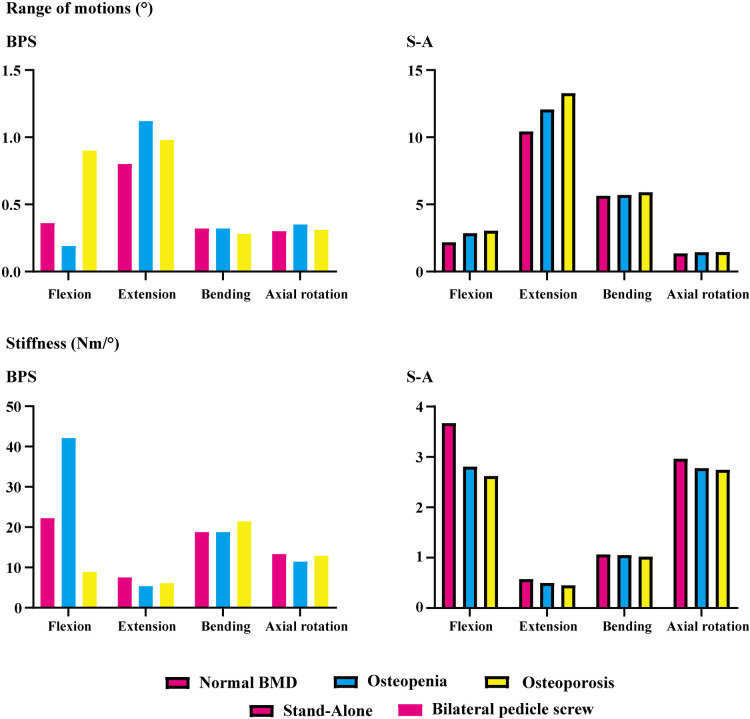
ROMs and stiffness of the motion segment caudal to the surgical segment (L5–S1).

ROMs and stiffness in adjacent segments were computed and recorded in the present study. Both cranial and caudal motion segments exhibited identical overall variation. Specifically, one step decrease in BMD aggravated motility compensation (i.e., ROMs increased and stiffness decreased with the decrease in the bony elastic modulus). Contrary to the common belief, motility compensation was greater on the caudal side than on the cranial side in both BPS and S-A models. Under the flexion loading condition, the most significant motility compensation was observed in S-A models. Compared to the model with normal BMD, ROMs increased by nearly 80% in both cranial and caudal side motion segments (Figures 2, 3).

Only a few exceptions were observed in osteopenia models under axial rotation loading conditions, in which ROMs decreased by 6.73% and 1.99% in BPS and S-A OLIF models, respectively, and decreased by 4.93% in the osteoporosis model with BPS fixation. In addition, under the extension loading condition of BPS models, ROMs increased by 47.35% and 15.99% in osteopenia and osteoporosis models, respectively; compared to osteoporosis models, this was the only loading condition in which the motility compensation was more severe in osteopenia models (Figures 2, 3).

### Instantly postoperative stability in the surgical segment

**Figure 4:**
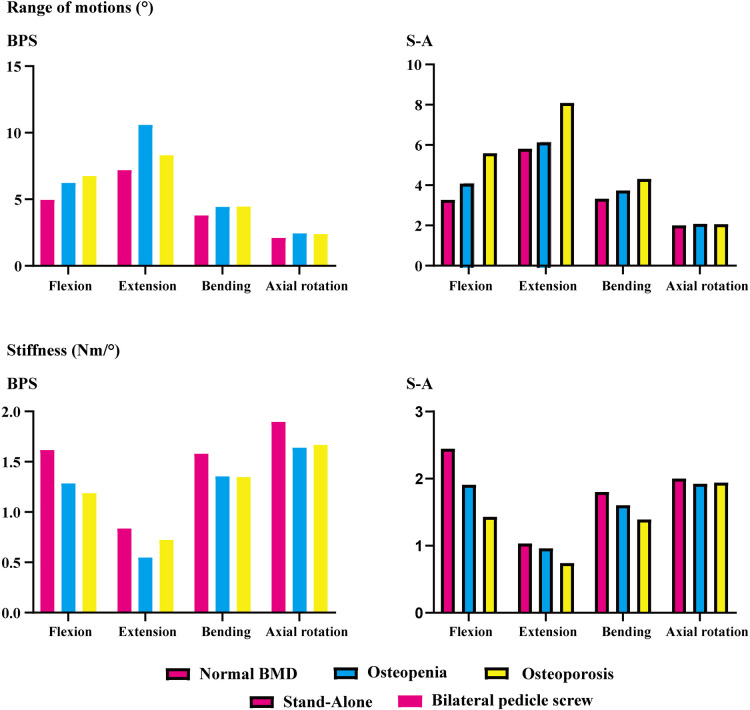
ROMs and stiffness of the surgical segment (L4–L5).

**Figure 5:**
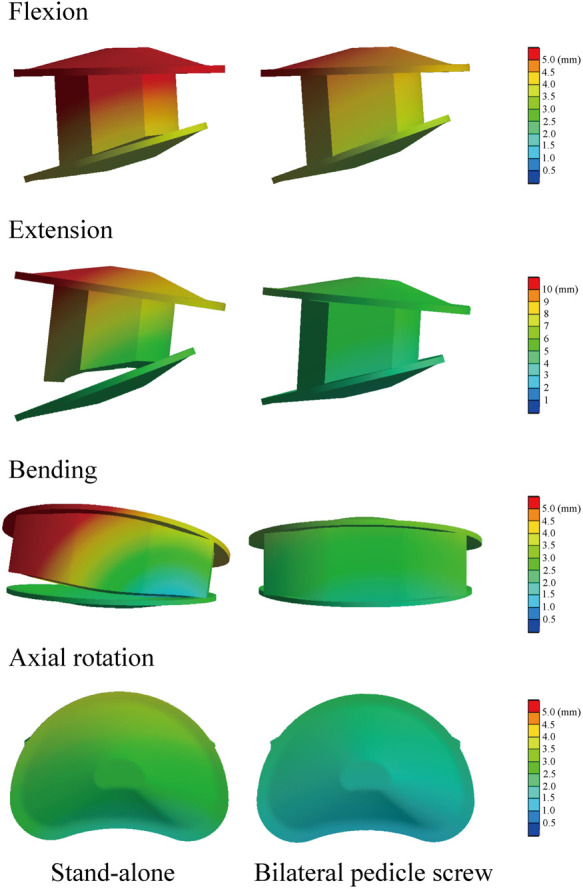
Nephograms for BEPs and the OLIF cage under different loading conditions.

BPS models showed smaller ROMs and higher stiffness in the surgical segment than S-A models. Under the flexion and axial rotation loading conditions, the ROMs of the S-A models were smaller than 3°, except for the osteoporosis model, whose ROM was slightly larger than 3° under the flexion loading condition. In contrast, poor instant postoperative stability of S-A models was observed under extension and lateral bending loading conditions, in which ROMs were larger than 5° under bending and even larger than 10° under extension loading conditions. As shown in the nephograms, apparent separations were observed between the BEPs and OLIF cage in the S-A models under extension and bending loading conditions (Figures 4, 5).

## Discussion

To investigate the biomechanical effects of BMD reduction on motility compensation of segments adjacent to the LIF operative segment, S-A OLIF and OLIF with BPS fixation were simulated in well-validated lumbosacral FE models with different BMDs. Computational results showed that the decrease in BMD leads to severe motility compensation of both cranial and caudal adjacent segments. This may be a reasonable explanation for the higher incidence of ASD in patients with poor BMD after LIF surgeries.

ASD is a widely reported complication after LIF surgeries. The recurrence of symptoms and the resulting reoperations adversely affect the clinical outcomes of patients. Biomechanical deterioration is an important cause of ASD (40, 41). Stress concentration and motility compensation are two types of biomechanical deterioration. Specifically, LIF surgery induces stress concentration in adjacent segments’ IVDs and facet cartilages of zygapophyseal joints (ZJs). These changes increase the incidence of annular tears and cause acceleration of DD and degenerative osteoarthritis of ZJs (6, 23). These pathological changes are common types of ASDs. Additionally, LIF surgeries increase the stiffness of the surgical segment (decrease ROMs under the same sizes of moments). Thus, during daily activities, the reduced ROMs of the surgical segment should be compensated by adjacent segments, which is also a common cause of ASD (3, 4, 9).

In the present study, the extent of motility compensation of adjacent segments steeply increased with the decrease in bony elastic modulus. This computational result partially explains the reason why patients with osteoporosis have a high risk of ASD biomechanically. Moreover, regular antiosteoporosis therapy is recommended in osteoporotic patients after LIF surgery. Generally, surgeons believe that this patient management strategy could reduce the incidence of surgical segment complications (e.g., screw loosening and cage subsidence). On the basis of current computational results, the significance of postoperative anti-osteoporosis has been further emphasized, and we believe that it could optimize clinical outcomes of patients by reducing the risk of both surgical segment and adjacent segment complications.

The pathological process of stress concentration in adjacent segments after LIF surgery has been widely reported. Previous studies have shown that the incidence rate of ASD in the segment cranial to the surgical segment was higher than that in the caudal segment, and the biomechanical mechanism of this phenomenon was the shorter force arm, resulting in higher grades of stress concentration of the cranial side IVD (5, 6, 15). However, the variation in motility compensation was inconsistent with stress concentrations. In the present study, the variation in motility compensation in the caudal segment was overall comparable to that in the cranial segment and even more pronounced in the caudal segment under some loading conditions. Considering the exact effect of motility compensation on the risk of ASD, the incidence of ASD on the caudal side adjacent segment should not be ignored in future clinical studies (Figures 2, 3).

The difference in instant postoperative stability between S-A OLIF and OLIF with BPS fixation was also compared. Consistent with the consensus, as the gold standard of AFD, BPS fixation provides excellent fixation stability (13, 42). However, the apparent separation between the OLIF cage and BEPs in the S-A models was considered for daily size moments (especially under extension and lateral bending loading conditions) (Figure 5). Therefore, although the BPS models show more severe motility compensation, we recommend using AFDs in OLIF patients to reduce the incidence of complications related to the separation between BEPs and the OLIF cage (e.g., cage migrations and nonunions). We also recommend restricting daily motions or AFDs (e.g., semirigid waistline) in S-A patients in the early postoperative period to reduce complication risk.

In conclusion, although no consensus was noted on the relationship between poor BMD and ASD incidence by computing stress concentration grades in adjacent segment IVDs and ZJs, a clear variation in motility compensation was observed in current models, and a reasonable explanation can be derived from the biomechanical perspective. Specifically, following the reduction in bony BMD, differences in stiffness between the LIF motion segment with insertional devices (e.g., OLIF cage, GB, and AFDs) and adjacent segment IVDs and more severe motility compensation can be deduced.

The conclusion of this study should be accepted only after acknowledging the following limitations. As an inherent defect of FE studies, the present study could not simulate *in vivo* biological and morphological changes during the interbody fusion process. Therefore, it is difficult to simulate the influence of BEP damage, cage subsidence, and screw loosening on adjacent segments of biomechanical environments. More significantly, spinal instability could induce *in vivo* self-adaptation mechanics, leading to the generation of osteophytes that affect local biomechanical environments, which was also ignored in this study (43, 44). We hope to address these limitations in future studies by further calibration and optimization of FE models.

## Conclusion

Poor BMD aggravates the motility compensation of the adjacent segment after S-A OLIF and OLIF with BPS fixation; this variation may increase the incidence of ASD. The S-A surgical method cannot provide instant postoperative stability; hence, daily motions of S-A patients should be restricted, or AFDs (e.g., semirigid waistline) should be used in the early postoperative period to avoid surgical segment complications.

## Data Availability

The original contributions presented in the study are included in the article/Supplementary Material, further inquiries can be directed to the corresponding author/s.
